# Cost‐effectiveness of swab versus tissue sampling for infected diabetic foot ulcers from the CODIFI2 randomised controlled trial

**DOI:** 10.1111/dme.15492

**Published:** 2025-02-27

**Authors:** Chris Bojke, Henrietta Konwea, E. Andrea Nelson, Sarah T. Brown, Colin C. Everett, Angela Oates, Michael Backhouse, Howard Collier, Joanna Dennett, Rachael Gilberts, Benjamin A. Lipsky, Michelle M. Lister, Jane E. Nixon, David Russell, Tim Sloan, Fran Game, the CODIFI2 Investigators, Ravikanth Gouni, Haroon Siddique, Gillian A Lomax, Joseph M Pappachan, Satyan Rajbhandari, David Russell, Mushtaqur Rahman, Ryan D’Costa, Biswa Mishra, Zoe Boulton, Rajiv Gandhi, Anna Goodman, Vasileios Tsadlidis, Kilimangalam Narayanan

**Affiliations:** ^1^ Faculty of Medicine and Health University of Leeds Leeds UK; ^2^ Glasgow Caledonian University Glasgow UK; ^3^ CTRU University of Leeds Leeds UK; ^4^ Leeds Beckett University Leeds UK; ^5^ Warwick Clinical Trials Unit University of Warwick Warwick UK; ^6^ University of Washington Seattle USA; ^7^ Nottingham University Hospitals NHS Trust Nottingham UK; ^8^ University of Nottingham Nottingham UK; ^9^ University Hospitals of Derby and Burton NHS Foundation Trust Derby UK

**Keywords:** antibacterial agents, cost‐effectiveness analysis, diabetic foot, quality‐adjusted life‐years, randomised controlled trial, wound sampling

## Abstract

**Aims:**

To compare the cost‐effectiveness of wound swabbing versus tissue sampling for infected diabetic foot ulcers.

**Methods:**

This multi‐centre, Phase III, prospective, unblinded, two‐arm parallel group, randomised controlled trial compared clinical (reported elsewhere) and economic outcomes of swab versus tissue sampling over a 52–104 week period.

Resource use was logged using case record forms and patient questionnaire at weeks 4, 12, 26, 39, 52 and 104, costed using laboratory and published sources from the UK NHS perspective, at 2021/2022 price‐year. EQ‐5D‐3L questionnaires issued at these time points were used to derive quality‐adjusted life‐years (QALYs).

To account for imbalances such as age, a regression‐based approach was used to estimate survival, expected costs and QALYs between the sampling arms. Available case analysis (ACA) and multiple imputation methods were applied for self reported missing data, and ACA for researcher‐collected data (survival, hospitalisations and antibiotic use). Probabilistic sensitivity analysis was used to assess the uncertainty of economic results.

**Results:**

We recruited 149 participants (75 swab, 74 tissue) from 21 UK sites, between 07 May 2019 and 28 April 2022 (last follow‐up 28 April 2023). Planned sample size was 730 participants, for 90% power to detect 12.5% difference in healing at 52 weeks, but the trial stopped early due to low recruitment.

Expected QALYs in the swab‐sampling arm were greater than in the tissue‐sampling arm at weeks 26, 52 and 104.

The cost of tissue sampling was greater than of swabbing when including antibiotics and hospitalisation. Swab sampling participants had higher QALYs and lower costs across weeks 26–52, reducing slightly by week 104.

**Conclusions:**

Because of higher costs, lower QALYs and lack of evidence of benefit, potentially due to the trial being underpowered, tissue sampling was dominated by wound swabbing in the cost‐effectiveness analysis.

**What's new?**

**What is already known?**

- Diabetic foot ulcer (DFU) guidelines recommend tissue sampling for culture of infection.
- Tissue samples, identify pathogens in more patients and report more pathogens than wound swabs.

**What is the key question?**

- Does tissue sampling produce different outcomes for quality of life (QALY) and cost‐effectiveness than swabbing in clinical practice?

**What this study found?**

- Lower QALY and higher costs for tissue sampling suggest it was not cost‐effective compared with swab sampling.

**What are the implications?**

- If replicated, these findings suggest clinicians may choose swabbing over tissue sampling for mild or moderately infected DFU with no effect on healing and reduced costs.

## 1 INTRODUCTION

The annual incidence of diabetic foot ulcers (DFUs) is 2%–10% of all people with diabetes mellitus.^1^, ^2^ These ulcers are susceptible to infection, with about 40% of recent onset cases being clinically infected at presentation.^3^ Prompt diagnosis and antibiotic treatment are needed to limit the progression of DFU infection and reduce the risk of lower extremity amputations and associated morbidity.

Pathogens causing infection can be detected in various types of wound samples. Swab sampling of the wound surface is a relatively quick, easy, non‐invasive, inexpensive and readily available method. Tissue sampling from the wound bed is a more invasive approach requiring special training, but is more likely to detect more pathogens, less likely to include colonising bacteria and better supports the growth of obligately anaerobic or fastidious organisms.^4^, ^5^, ^6^, ^7^, ^8^, ^9^ Thus, tissue samples are often characterised as providing ‘better information’ which should lead to improvement in treatment decisions and outcomes, but this has not, to date, been demonstrated in any clinical comparisons of the approaches.

Tailoring of antibiotic therapy, post‐empirical therapy, based on tissue, rather than swab, sampling, might lead to more rapid resolution of infection, and potentially quicker healing, improved prescribing and antimicrobial stewardship.^10^, ^11^ Conversely, tissue sampling's higher yield compared with wound swabbing could increase the likelihood of unnecessary or overly broad antibiotic prescribing, leading to increased costs, adverse effects and development of antibiotic resistance.

CODIFI2 (ISRCTN74929588; registered 8 January 2019) was a multi‐centre, Phase III, prospective, 2‐arm parallel group, randomised controlled trial, comparing the effects on time to healing of DFU clinically assessed as having a mild or moderate infection, using two different sampling techniques (swab vs. tissue sampling), with blinded outcome assessment. Secondary outcomes, including cost‐effectiveness, were also compared. This paper reports the health economics analysis of the data, while a companion paper reports the details of the trial methodology and results for the clinical outcomes.

## 2 METHODS

A within‐trial cost‐utility analysis was conducted to compare the resource use, costs and health benefits incurred in both the tissue and swab sampling arms over at least 52 weeks, from the perspective of the UK National Health Service (NHS) and Personal Social Services (PSS).^12^, ^13^ Results are reported for weeks 26, 52 and as a sensitivity analysis at week 104.

### 2.1 Participants and Setting

CODIFI2 recruited participants (aged ≥18 years) from UK NHS secondary care and community clinics providing multidisciplinary team DFU services, who had DFUs clinically suspected to have mild or moderate soft tissue infection.

### 2.2 Interventions

The interventions compared were policies of swab sampling or tissue sampling, both at initial presentation and at follow‐up sampling during the trial. Participants were to have all DFUs sampled by the allocated method, with any deviations recorded. All samples were delivered to local microbiology laboratories for routine culture and sensitivity (C&S) processing as soon as possible after collection.

#### 2.2.1 Resource use and costs

Healthcare resource use (HRU) within the analysis included the type and number of samples (baseline and follow‐up), hospitalisations (for amputations, revascularisation, foot surgery and other related causes) and antibiotic prescriptions based on case record forms (CRF). These data were collected continuously over the trial period. Contacts with healthcare professionals (HCPs) such as general practitioners (GPs), practice and district nurses, non‐admitted hospital attendance (foot clinic, outpatients or emergency department) captured via the HRU questionnaire administered at weeks 4, 12, 26, 39, 52 and 104, asking about use in the previous 4 weeks. A summary of the resource use and cost data is in Table 1.

**TABLE 1:** Unit costs of sampling and community care.

Resource	Data source	Unit Cost (£)	Cost source
Sampling	Baseline and follow‐up CRFs		
Tissue		20	Local laboratory
Swab		12	Local laboratory
Bone	Follow‐up CRF	124^a^	National Schedule of Costs 2015/2016, inflated to 2021/2022
HCP contacts and non‐admitted visits	HRU Questionnaire		
GP		38^b^	PSSRU 2022
Practice nurse		13^c^	PSSRU 2022
District nurse		54	National Schedule of Costs 2021/2022
Foot clinic		93	
Outpatient		183	
A&E		144	

Abbreviations: A&E, Accident and Emergency; CRF, Case Report Form; GP, General Practitioner; HRU, Healthcare resource use; PSSRU, Personal and Social Services Research Unit.

^a^ HRG codeYH31Z: Image‐guided biopsy of bone lesion.

^b^ Per patient contact of 9.22 min; with qualifications, excluding direct care cost.

^c^ £52 per hour with qualifications; assumed duration of 15.5 min based on last available PSSRU (2016) data.

The costs of sampling methods were provided by local microbiology laboratories. Other costs were from published sources including secondary care visits,^12^ primary care visits^13^ and antibiotics,^14^ with all costs based on 2021/2022 prices.

Healthcare resource group (HRG) codes for hospitalisation events were manually assigned to reported data and used to calculate the final weighted average cost for each event^12^, ^15^ (see Table 2). To accommodate systematic differences in length of stay in resource use calculations, 2015/2016 reference costs, adjusted for inflation, were used for admissions costs, as this was the last available source of excess bed‐days and length of stay data^13^, ^16^. Where more than one admission event was recorded for the same dates, the more expensive event was selected. For the estimation of amputation costs, assumptions from a cost of DFU study were applied.^17^

**TABLE 2:** Resource use and cost of hospitalisation for serious adverse events (SAEs) requiring admission.

Events	Cost per episode (£)	Average length of stay (days)	Cost of excess bed days (£)	Notes/Assumptions
Amputations (major)^a^	11,723	19	313	Weighted average of codes YQ22A, B: Amputation of single limb with CC scores 0–10+
Amputations (minor)^a^	5385	9	328	Weighted average of codes YQ26A, B, C: Single, amputation stump or partial foot amputation procedure, for diabetes or arterial disease, with CC Score 0–8+
Revascularisation^a^	8329	7	368	Weighted average of codes YQ11A‐C: Single open procedure on blood vessel of lower limb with imaging intervention, with CC Score 0–7+, codes YQ12A‐D: Single open procedure on blood vessel of lower limb with CC Score 0–11+
Osteotomy; IPJ^a^	2506	2	373	Weighted average of codes HN33 and HN34: Intermediate and major foot procedures for non‐trauma, Score 0–4+
Surgical debridement; incision and drainage of abscess in the foot; tenotomy^a^	817	1	0	Code HN35: Minor foot procedures for non‐trauma
Arthroplasty^a^	3969	2	389	Weighted average of codes HN32A‐C Very major foot procedures for non‐trauma
Osteomyelitis^a^	3905	8	314	Weighted average of codes HD25D‐H: Infections of bones or joints with CC Score 0–13+
Removal of external frame (fixation)^a^	2034	2	357	Weighted average of codes HN33 and HN34: Intermediate and major foot procedures for non‐trauma, Score 0–4+
Gastrocnemius release^a^	2034	2	357	Weighted average of codes HN33 and HN34: Intermediate and major foot procedures for non‐trauma, Score 0–4+
Cellulitis^a^	1791	6	1791	Weighted average of codes within JD07: Skin disorders with/without interventions
Other unclassified causes^a^	2637	7	295	Weighted average of codes within KB03: Diabetes with lower limb complications
Post‐amputation cost^b^	529	‐	‐	weighted average of codes REHAB 1–3: Rehabilitation for amputation of limb

^a^ National Schedule of Costs 2015/2016, uprated to 2021/2022.

^b^ National Schedule 2021/2022.

Details of antibiotic prescriptions for participants were taken from the site‐completed antibiotic diary. Where prescribing information, such as duration of use, was missing for any antibiotic prescribed, assumptions were made based on the NICE prescribing guidelines for DFU infection or minimum expected dosage requirements stated in the British National Formulary (BNF)^14^ or individual summary of product characteristics (SPC) (see Table 3).

**TABLE 3:** Antibiotic costs.

Antibiotic name and pharmaceutical form	Average cost per unit form
Amoxicillin 500 mg capsule	£0.21
Benzylpenicillin 600 mg vial	£2.89
Ceftazidime 2 g	£16.43
Ceftriaxone 250 mg powder for solution for infusion vial	£2.37
Ceftriaxone 1 g powder for solution for infusion vial	£8.61
Ceftriaxone 2 g powder for solution for infusion vial	£19.00
Clindamycin 300 mg capsule	£1.03
Clindamycin 150 mg/mL ampoule	£8.77
Clarithromycin 500 mg tablet	£0.65
Clarithromycin 500 mg vial	£10.85
Ciprofloxacin 500 mg tablet	£0.25
Co‐amoxiclav 500 mg/125 mg tablet	£0.39
Co‐amoxiclav 500 mg/100 mg powder for solution for injection vial	£1.30
Co‐trimoxazole 160 mg/800 mg tablet	£0.25
Doxycycline 100 mg capsule	£0.16
Ertapenem 1 g powder vial	£31.65
Erythromycin 500 mg tablet	£0.45
Erythromycin 1 g powder vial	£22.46
Flucloxacillin 500 mg capsule	£0.16
Flucloxacillin 500 mg vial	£9.66
Sodium fusidate (fusidic acid) 500 mg vial	£21.95
Gentamicin (as Gentamicin sulfate) 40 mg per 1 mL vial	£1.38
Linezolid 600 mg capsule	£35.48
Meropenem 500 mg	£9.87
Metronidazole 400 mg	£0.20
Metronidazole 5 mg per 1 mL infusion bag	£3.49
Phenoxymethylpenicillin 250 mg tablets	£0.12
Piperacillin 4 g/Tazobactam 500 mg	£12.18
Rifampicin 600 mg	£9.20
Teicoplanin 200 mg	£6.55
Tigecycline 50 mg	£29.08
Trimethoprim 200 mg	£0.27

#### 2.2.2 Health‐related quality of life (HRQoL)

Participants' responses to the EuroQol—5‐Dimension (EQ‐5D‐3L) questionnaire at baseline, weeks 4, 12, 26, 39, 52 and 104 post‐randomisation were converted to utility scores using the standard UK general population time trade‐ off tariff values.^18^ To accommodate patient deaths, a parametric survival model was estimated and the expected HRQoL in terms of quality‐adjusted life‐years (QALYs) under each treatment was calculated by multiplying the probability of survival by the conditional expectation of HRQoL. QALYs were subsequently estimated using the area under the curve calculation.

#### 2.2.3 Statistical analysis of patient‐level data for economic evaluation

All data manipulation and analysis for the economic evaluation were conducted using SAS 9.4. To account for baseline imbalances such as age, a regression‐based approach was taken for the estimation of survival, expected costs and HRQoL between the sampling arms. In all regressions, the same set of explanatory variables was used: mean‐adjusted age, sex, number of ulcers, ulcer area and duration. The following regression models were estimated.

- Survival: Log‐normal parametric survival model (best fitting parametric model according to AIC and BIC criteria).
- EQ‐5D HRQoL: Linear model with random effects.
- Probability of hospitalisation: Ordinal logistic regression (no hospitalisation, other hospitalisation minor amputation or revascularisation and major amputation).
- Probability of antibiotic prescription: Logistic regression.
- Other NHS cost: Linear model with random effects.

Random effects models to account for unobserved patient heterogeneity were attempted for the logistic models but they would not converge. Costs for hospitalisations and antibiotic use were determined by multiplying the regression‐modelled probability of a cost occurring by unit cost of event.

Other cost data referred to costs in the preceding 4 weeks, regardless of time point. It was assumed that observations taken at weeks 12, 26, 39, 52 and 104 were representative of the whole period since the last observation and were extrapolated as such. Because hospitalisations and antibiotic use cost data were collected continuously, no simplifying assumption was needed.

Due to the use of non‐linear models a corrected group prognosis—CGP^19^ was applied to produce population averages. CGP works by combining the observed individual patient characteristics (including estimate of patient random effect) and regression results to estimate outcomes over time for each patient under the counterfactual treatment conditions. These outcomes were averaged over all patients, and the difference in these average outcomes were used in the analysis.

#### 2.2.4 Missing data

Missing data are a common issue in trials with increased attrition over time. For CODIFI2, this issue was exacerbated by the early termination of the trial. This substantially increases the number of missing data points, for example, by limiting 104‐week follow‐up data to patients starting in the first year where EQ‐5D data from 51/75 (68%) of the swab arms and 56/74 (76%) of the tissue arm were missing (see Table 4). As the main driver of such attrition was administrative (early trial termination), the common multiple imputation solution assumption of missing at random (MAR) is thought to be more tenable. As such both available case analysis (ACA) and multiple imputation (MI) solutions were adopted for self reported missing data (EQ‐5D and other costs). Fully conditional specification regression models with patient characteristics and all outcomes were used in MI, with 100 iterations sampled.

**TABLE 4:** Distribution of observed ED‐5D data and estimated utilities.

Visit Week	Swab sampling						Tissue sampling					
	Total	Missing	Dead	Completed EQ‐5D questionnaire	Average EQ‐5D utility	Standard error EQ‐5D utility	Total	Missing	Dead	Completed EQ‐5D questionnaire	Average EQ‐5D utility	Standard error EQ‐5D utility
0	75	5 (7%)	0 (0%)	70 (93%)	0.511	0.043	74	3 (4%)	0 (0%)	71 (96%)	0.567	0.036
4	75	14 (19%)	0 (0%)	61 (81%)	0.551	0.044	74	14 (19%)	0 (0%)	60 (81%)	0.637	0.038
12	75	26 (35%)	0 (0%)	49 (65%)	0.608	0.050	74	24 (32%)	0 (0%)	50 (68%)	0.545	0.046
26	75	35 (47%)	3 (4%)	37 (49%)	0.529	0.068	74	28 (38%)	0 (0%)	46 (62%)	0.583	0.048
39	75	38 (51%)	6 (8%)	31 (41%)	0.404	0.070	74	44 (59%)	1(1%)	29 (39%)	0.474	0.068
52	75	37 (49%)	8 (11%)	30 (40%)	0.469	0.065	74	39 (53%)	3 (4%)	32 (43%)	0.400	0.057
104	75	51 (68%)	17 (23%)	7 (9%)	0.142	0.067	74	56 (76%)	7 (9%)	11 (15%)	0.370	0.089

#### 2.2.5 Cost‐effectiveness analysis

Discounting of both costs and utilities was applied at 3.5% for the second year in the sensitivity analysis. Cost and QALY data were combined to calculate an incremental cost‐effectiveness ratio (ICER). The Incremental net monetary benefit (INMB) and incremental net health benefit (INHB) statistics were subsequently derived from the ICER calculation. The limit of £20,000–£30,000 threshold (λ) was applied such that, for a given λ, an intervention with a positive mean incremental benefit (INMB or INHB >0) should be adopted.

#### 2.2.6 Probabilistic sensitivity analysis (PSA)

PSA was conducted by directly drawing samples from the regression results and the associated variance–covariance matrices to capture the correlation between parameter estimates. One thousand iterations of new regression parameters were drawn and applied to the model to obtain expected costs and QALYs for each individual under both treatment options. Results were averaged across the population to estimate expected costs and QALYs.

## 3 RESULTS

A total of 149 participants were randomised between 7 May 2019 and 28 April 2022, with trial closure 3 May 2022. Demographic and baseline characteristics of participants are summarised in Table 5. The groups were similar in most respects, except that participants in the swab group were on average 6 years older than those in the tissue group (mean [range] 65.7 [32–93] and 59.7 [31–86] for swab vs tissue, respectively) and had a slightly higher proportion of men [86.7% vs. 78.4%], respectively.

**TABLE 5:** Baseline characteristics of trial participants.

	Swab sampling (*n* = 75)	Tissue sampling (*n* = 74)	Total (*n* = 149)
Age, years	65.7 (11.39)	59.7 (12.98)	62.7 (12.54)
[Minimum, maximum]	[32, 93]	[31, 86]	[31, 93]
Gender, men	65 (86.7%)	58 (78.4%)	123 (82.6%)
Ethnicity, white	72 (96.0%)	72 (97.3%)	144 (96.6%)
Type II Diabetes	68 (90.7%)	63 (85.1%)	131 (87.9%)
Duration of diabetes (years) median [IQR]	15.0 [10.0–24.0]	17.0 [11.0–21.0]	16.0 [10.0–22.0]
HbA1c, mmol/mol	70.1 (22.97)	73.3 (24.09)	71.7 (23.49)
Current treatment for diabetes			
Oral hypoglycaemic agent	52 (69.3%)	45 (60.8%)	97 (65.1%)
Insulin	37 (49.3%)	47 (63.5%)	84 (56.4%)
Other non‐insulin injectables	8 (10.7%)	3 (4.1%)	11 (7.4%)
Diet alone	9 (12.0%)	2 (2.7%)	11 (7.4%)
DFU on both feet	9 (12.0%)	8 (10.8%)	17 (11.4%)
More than one ulcer at baseline	21 (28.0%)	20 (27.0%)	41 (27.5%)
Index DFU initial (non‐recurrent)	59 (78.7%)	59 (79.7%)	118 (79.2%)
Index DFU Aetiology			
Neuro‐ischaemic	10 (13.3%)	7 (9.5%)	17 (11.4%)
Ischaemic	‐	2 (2.7%)	2 (1.3%)
Neuropathic	64 (85.3%)	65 (87.8%)	129 (86.6%)
No neuropathy or ischaemia (unusual presentation)	1 (1.3%)	‐	1 (0.7%)
Index DFU Located on forefoot (+/− digits)	63 (84.0%)	61 (82.4%)	124 (83.2%)
Duration of index DFU (months) Median [IQR]	1.0 [0.5–3.0]	2.0 [0.5–4.0]	1.0 [0.5–4.0]
Index Ulcer Grade			
Grade 1—Superficial full‐thickness	46 (61.3%)	52 (70.3%)	98 (65.8%)
Grade 2—Deep ulcer, penetrating to below dermis	26 (34.7%)	15 (20.3%)	41 (27.5%)
Grade 3—Affecting all layers, including bone and/or joint	3 (4.0%)	7 (9.5%)	10 (6.7%)
Index DFU area (cm^2^) Median [IQR]	2.2 [0.7–4.7]	1.1 [0.5–3.1]	1.3 [0.6–3.8]
Infection Classification			
Mild	53 (70.7%)	46 (62.2%)	99 (66.4%)
Moderate	22 (29.3%)	27 (36.5%)	49 (32.9%)
Grade 4	‐	1 (1.4%)	1 (0.7%)
Prior treatments			
Both antimicrobial/antiseptic dressings and antibiotics (any indication)	9 (12.0%)	9 (12.2%)	18 (12.1%)
Antibiotics (any indication) only	12 (16.0%)	10 (13.5%)	22 (14.8%)
Antimicrobial/antiseptic dressings only	17 (22.7%)	15 (20.3%)	32 (21.5%)
Neither	37 (49.3%)	40 (54.1%)	77 (51.7%)

*Note*: Values are either mean (standard deviation) or *n* (percentage), unless otherwise stated.

### 3.1 Regression analysis of survival, HRQoL and costs

The full results of the regression analyses can be found in Tables S1 and S2.

Expected (mean) survival in the tissue sampling arm was greater than that in the swab sampling arm, after regression correcting for differences in age between groups, with expected survival at 0.96 and 0.85 with tissue sampling at 52 and 104 weeks and 0.90 and 0.72 with swab sampling. After accounting for age, the difference was not statistically significantly different.

In general, HRQoL for those alive was expected to be higher with swab sampling relative to tissue sampling at all time points up to 52 weeks. None of these differences were statistically significant except at week 52 where HRQoL was expected to be 0.15–0.2 higher using swab sampling, depending on whether the ACA or MI data are used. At week 104, the picture reversed with HRQoL was estimated to be higher with tissue sampling, though the result was not statistically significant. Relative to ACA, MI had the impact of increasing the differences in favour of swab at weeks 39 and 52.

Combining survival and conditional HRQoL expectations brings together two somewhat contradictory drivers of patient benefit with survival being higher with tissue sampling, but conditional HRQoL generally being higher with swab sampling. Over 52 weeks, the expected QALY difference is 0.04 (ACA) to 0.11 (MI) QALYs in favour of swab sampling. For 104 weeks, the differences are 0.05 and 0.19 QALYs, respectively.

For the cost regressions, the consistent finding is that excluding differences in the costs of testing, there are generally no consistent and significant differences between costs of treatments over time. Only the results for antibiotic cost difference between the two arms was statistically significant, with costs 82% greater in the tissue arm compared to the swab sampling arm (exp [0.602], *p* = 0.014).

### 3.2 Base case reporting

Given the use of non‐linear models and the uncertainty caused by analysing a small sample, the PSA mean results are preferred to the deterministic results as the base case analysis. Similarly, the MI method for accommodating missing data is preferred and, given the amount of missing data at week 104, the base case analysis was MI PSA set of data assessed at week 52.

The mean modelled cost and QALYs per resource group at weeks 26, 52 and 104 using MI PSA are shown on Table 6. This shows that the total expected costs in the tissue sampling arm were greater compared to the swab sampling arm at each of these time points. Conversely, all QALYs in the swab sampling arm were greater than the tissue sampling arm. All combinations of analyses are presented on Table 7, with results which are substantively consistent across all choices of methods and time frame.

**TABLE 6:** Cost‐effectiveness results (expected outputs)—base case analysis.

	Antibiotics costs		Hospitalisation costs		Other costs		Total			QALYs		
Weeks	Tissue	Swab	Tissue	Swab	Tissue	Swab	Tissue	Swab	Difference	Tissue	Swab	Difference
26	£393	£320	£6016	£5414	£2613	£1924	£9042	£7670	£1371.68	0.274	0.286	−0.013
52	£549	£446	£8659	£8089	£5148	£3989	£14,376	£12,535	£1840.96	0.499	0.535	−0.036
104	£779	£671	£12,561	£12,805	£8475	£8472	£21,835	£21,960	‐£124.83	0.899	0.951	−0.052

Abbreviations: Other, Sampling, HCP contacts and non‐admitted care.

**TABLE 7:** Expected cost‐effectiveness results of tissue versus swab sampling.

	ACA—Deterministic Estimations										
	QALYs			Costs			At £20 k per QALY gained		At £30 k per QALY gained		
Week	Tissue	Swab	Difference	Tissue	Swab	Difference	INHB	INMB	INHB	INMB	ICER
26	0.27	0.29	−0.01	£10,256	£8651	£1605	−0.09	‐£1859	−0.07	‐£1986	Dominated
52	0.50	0.53	−0.04	£15,820	£13,514	£2306	−0.15	‐£3030	−0.11	‐£3392	Dominated
104	0.90	0.95	−0.05	£23,349	£23,284	£65	−0.06	‐£1109	−0.05	‐£1631	Dominated

	ACA—PSA means										
	QALYs			Costs			At £20 k per QALY gained		At £30 k per QALY gained		
Week	Tissue	Swab	Difference	Tissue	Swab	Difference	INHB	INMB	INHB	INMB	ICER
26	0.27	0.29	−0.01	£8998	£7674	£1324	−0.08	‐£1574	−0.06	‐£1699	Dominated
52	0.50	0.53	−0.03	£14,393	£12,352	£2041	−0.14	‐£2737	−0.10	‐£3085	Dominated
104	0.89	0.94	−0.05	£21,534	£21,604	‐£70	−0.05	‐£936	−0.05	‐£1439	N/A

	MI—Deterministic estimations										
	QALYs			Costs			At £20 k per QALY gained		At £30 k per QALY gained		
Week	Tissue	Swab	Difference	Tissue	Swab	Difference	INHB	INMB	INHB	INMB	ICER
26	0.28	0.30	−0.03	£10,289	£8657	£1632	−0.11	‐£2138	−0.08	‐£2391	Dominated
52	0.46	0.56	−0.11	£15,767	£13,694	£2073	−0.21	‐£4175	−0.17	‐£5226	Dominated
104	0.75	0.94	−0.19	£23,611	£23,639	‐£28	−0.19	‐£3736	−0.19	‐£5618	N/A

	MI—PSA means										
	QALYs			Costs			At £20 k per QALY gained		At £30 k per QALY gained		
Week	Tissue	Swab	Difference	Tissue	Swab	Difference	INHB	INMB	INHB	INMB	ICER
26	0.26	0.30	−0.03	£9042	£7670	£1372	−0.10	‐£2042	−0.08	‐£2377	Dominated
52	0.46	0.55	−0.09	£14,376	£12,535	£1841	−0.18	‐£3617	−0.15	‐£4505	Dominated
104	0.75	0.88	−0.12	£21,835	£21,960	‐£125	−0.12	‐£2341	−0.12	‐£3574	N/A

Abbreviations: ACA, Available case analysis; ICER, Incremental cost‐effectiveness analysis; INHB, Incremental net health benefit; INMB, Incremental net monetary benefit; MI, Multiple imputation; QALY, Quality‐adjusted life‐years; PSA, Probabilistic sensitivity analysis.

**FIGURE 1:**
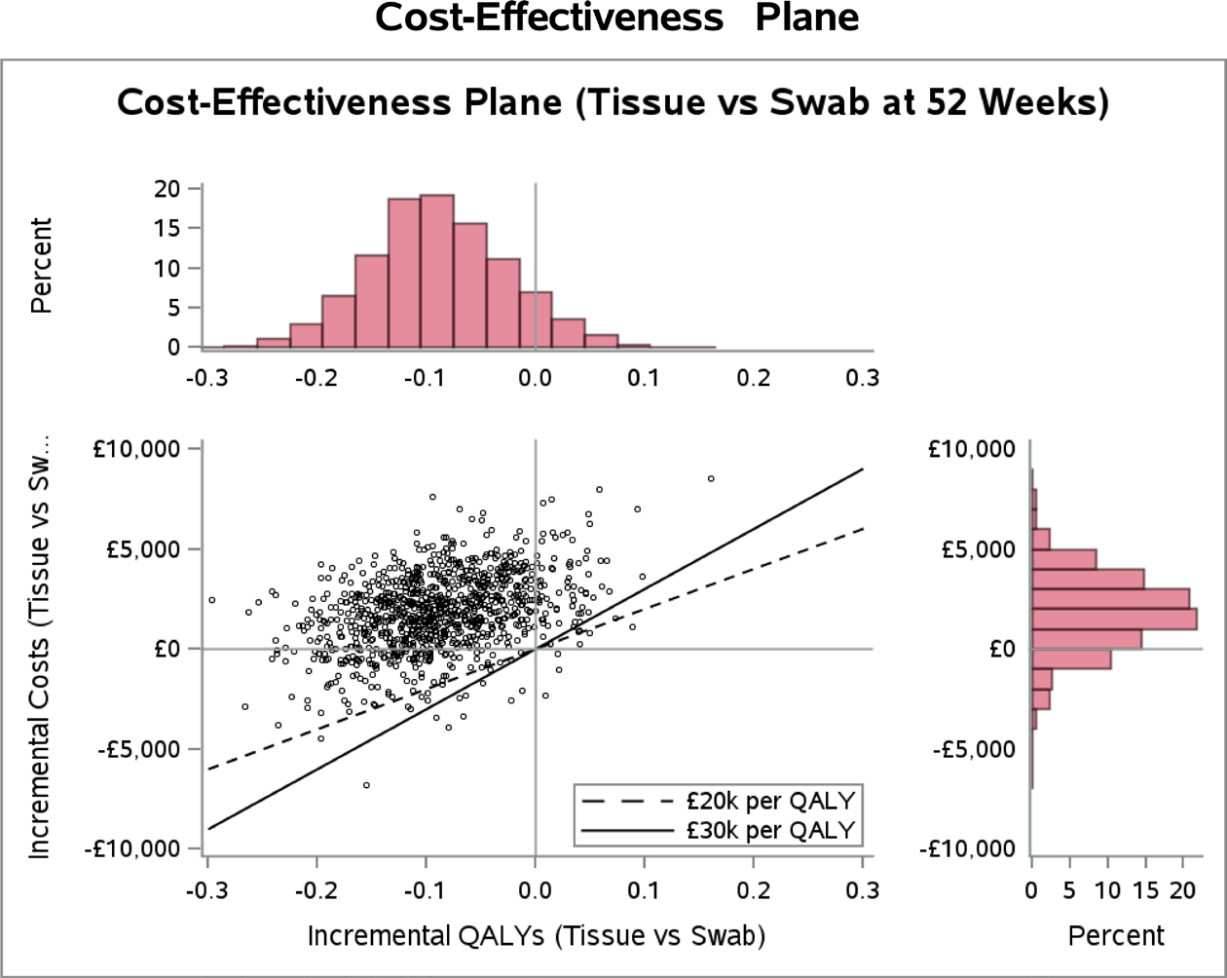
Cost‐effectiveness plane for MI PSA Week 52 analysis.

**FIGURE 2:**
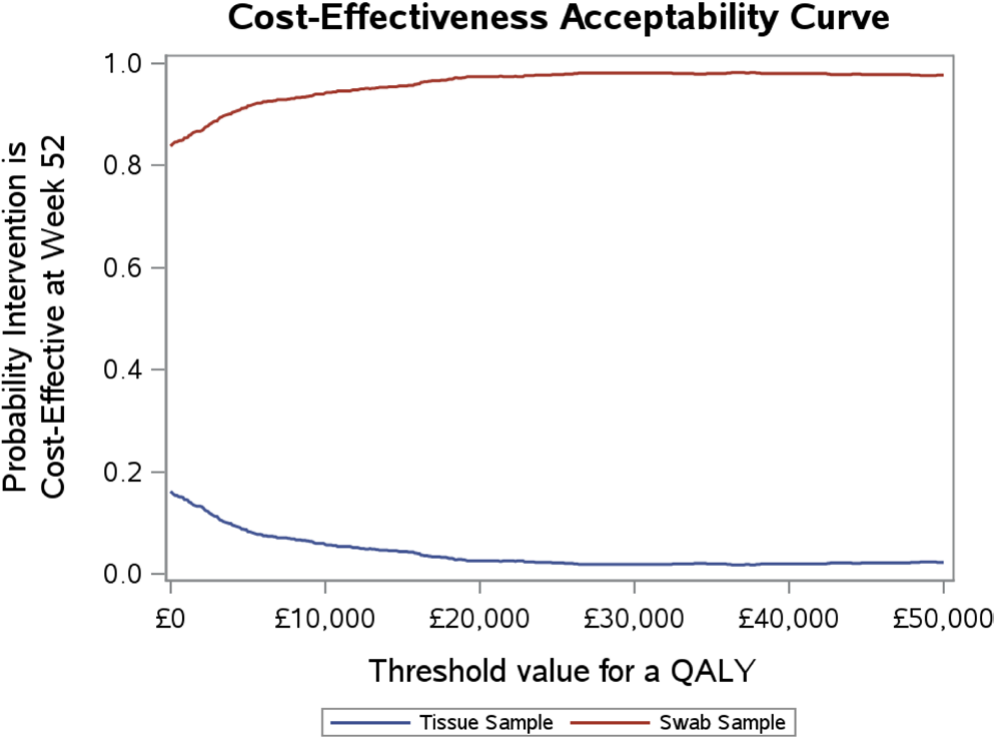
Cost‐effectiveness acceptability for MI PSA Week 52 analysis.

The base case results are shown as plots on a cost‐effectiveness plane in Figure 1, and construction of a cost‐effectiveness acceptability curve (CEAC) over the willingness to pay for a QALY range of £0–£50,000 is shown in Figure 2. The majority of the sampled joint distribution is in the north‐west quadrant (more costly, less beneficial for tissue) of the plane, while the probability of being cost‐effective becomes less with increasing values of λ reflecting the greater emphasis on QALY outcomes (Figure 2).

## 4 DISCUSSION

Despite recommendations in various guidelines that specimens for culture from infected DFUs should be collected by tissue sampling,^20^ in the great majority of cases, they are still collected by swabbing. The clinical results (summarised in our companion paper) concluded that there was no evidence of a difference in time to healing of infected DFU between the two sampling methods. Although expected survival was greater with tissue sampling in the present study, the overall QALY expectation was greater with swab sampling at all time points. In addition, tissue sampling was more costly than swab sampling assessed at weeks 26 and 52. At week 104, it was cost‐saving, but not to the extent that it became cost‐effective. Overall, this study suggests that tissue sampling, compared to swab sampling, for these patients is not cost‐effective.

The largest contribution to the QALY differences is driven by results at week 52; although HRQoL is expected to be higher with tissue sampling at week 104, it is not sufficient to outweigh the superior QALY with swab sampling over the preceding time points. The QALY gap is larger with MI than with ACA, again primarily due to increasing the incremental gap at week 52. The finding that QALY expectations are greater with swab sampling is consistent across all variations of analysis, including with different combinations of PSA versus deterministic and MI versus ACA.

The results of this study show that the expected cumulative cost of tissue sampling was greater than that of swab sampling evaluated at weeks 26 and 52. This was mainly due to increased costs of hospitalisations, although PSA analysis show these differences to be very uncertain. As observed with the HRQoL analysis, the expected costs generated post‐week 52 are expected to be less for tissue sampling. However, the overall expected costs at week 104 indicate that tissue sampling generates marginally smaller costs over the whole period.

In terms of variation across reporting methods, there is almost no difference between PSA and deterministic reporting choices, but the MI method increases the incremental QALY gap in favour of swab sampling. The difference in choice of time frame is more sensitive with the 104‐week analysis, suggesting that the direction of benefits changes after week 52 but not to the extent that the conclusions over the whole period are changed.

The week 104 results are based on a very small sample size, but raises the question whether conclusions may have been different if a longer time had been assessed. One of the main drivers of the difference in QALY outcomes is that the estimated (but not statistically significant) improved survival expectations of tissue sampling become greater over time.

Despite the small sample and generally non‐significant regression results, the PSA reveals surprisingly definite conclusions on the cost‐effectiveness argument. This is mostly due to the strong significant finding in HRQoL differences at week 52.

### 4.1 Strengths and weaknesses of the economic evaluation

Strengths of this evaluation include the use of detailed health economics through costing of real‐time resource use and utility estimation based on responses from the target population. This allowed us to carry out an assessment of the cost‐effectiveness of the two approaches to sampling. We have not been able to identify any previous trials of wound sampling approaches with cost‐effectiveness outcomes. In addition, this evaluation demonstrated the benefit of using appropriate techniques for handling missing data and characterising uncertainty around the cost‐effectiveness of tissue sampling.

Challenges for this study included the difficulty in utilising patient‐reported resource use, such as antibiotic prescriptions and equipment, due to lack of clarity in the raw data. However, more reliable data were obtained from the researcher‐led record review following participant contact.

### 4.2 Unanswered questions and future research

Studies with a longer follow‐up period and larger sample size might demonstrate a reverse of our findings that would favour tissue sampling, given the observed reverse in trend (mainly for costs) at week 104 for this arm, although the difficulties of very long follow‐up are considerable in this group, due to poor prognosis.^21^

## 5 CONCLUSIONS

Despite tissue sampling being widely recommended as being superior to swab sampling for the quality of information provided on wound microbiology, no previous trials have compared clinical and cost‐effectiveness of the two approaches. In addition to the lack of evidence of benefit observed with tissue sampling in CODIFI2, this within trial analysis found that tissue sampling was associated with higher costs and lower QALYs, making it not cost‐effective when compared with swab sampling.

## AUTHOR CONTRIBUTIONS

**E Andrea Nelson**: SC to conception and design, analysis and interpretation of data and drafting and guarantor. **Fran Game**: SC to conception and design, analysis and interpretation of data and drafting and review. **Jane E Nixon**: SC to conception and design and interpretation of data and reviewing critically for intellectual content. **Sarah T Brown**: SC to conception and design, analysis and interpretation of data and drafting and review.**Colin C Everett**: SC to conception and design, analysis and interpretation of data and drafting and review. **Howard Collier**: SC to acquisition of data and reviewing. **Angela Oates**: SC to conception and design, analysis and interpretation of data and drafting and reviewing. **Henrietta Konwea**: SC to analysis, interpretation of data and health economics and drafting and reviewing. **Chris Bojke**: SC to analysis, interpretation of data and health economics, drafting and reviewing and guarantor. **Joanna Dennett**: SC to acquisition of data and Reviewing. **Rachael Gilberts**: SC to design and acquisition of data and reviewing. **David Russell**: SC to conception and design, acquisition of data and reviewing. **Tim Sloan**: SC to conception and design, analysis and interpretation of data and reviewing. **Michael Backhouse**: SC to conception and design and reviewing. **Michelle M Lister**: SC to conception and design, analysis and interpretation of data and reviewing.

## FUNDING INFORMATION

This study was funded by the UK National Institute for Health Research Health Technology Assessment Programme. It was a commissioned call for research (reference 16/163/04); hence, the funders specified the study population and sample, the intervention and the primary outcomes. The sponsor was the University of Leeds. The sponsor and funder were not involved in the design of the study; the collection, analysis and interpretation of data; writing the report; and did not impose any restrictions regarding the publication of the report.

## CONFLICT OF INTEREST STATEMENT

E.A.N., T.J.S., F.G., R.G., H.K., D.R., A.O., M.B. and B.A.L. declare no conflicts of interest.

## TRIAL REGISTRY NUMBER

ISRCTN74929588.

**CODIFI2** Clinical Investigators were: Dr. Ravikanth Gouni, Nottingham University Hospital NHS Trust, Principal Investigator/Consultant in Diabetes and endocrinology; Haroon Siddique, The Dudley Group NHS Foundation Trust, Principal Investigator/Consultant in Diabetes and Endocrinology; Gillian A Lomax, East Lancashire Hospitals NHS Trust, Principal Investigator/Extended Scope Practitioner; Professor Joseph M Pappachan, Lancashire & South Cumbria NHS Foundation Trust, Principal Investigator/Consultant & Research Lead in Endocrinology; Dr. Satyan Rajbhandari, Lancashire & South Cumbria NHS Foundation Trust, Consultant; Mr. David Russell, Leeds Teaching Hospitals NHS Trust, Associate Professor and Honorary Consultant Vascular Surgeon; Dr. Mushtaqur Rahman, London North West University Healthcare NHS Trust, Principal Investigator/Consultant; Dr. Ryan D'Costa, Mid Yorkshire Hospitals NHS Trust, Principal Investigator/Consultant; Dr. Biswa Mishra, Northern Care Alliance NHS Foundation Trust, Principal Investigator/Consultant Physician; Zoe Boulton, Royal Devon and Exeter University Healthcare Trust, Principal Investigator/Lead Diabetes Podiatrist; Dr. Rajiv Gandhi, Sheffield Teaching Hospital NHS Foundation Trust, Principal Investigator/Consultant; Dr. Anna Goodman, Guy's and St Thomas' NHS Foundation Trust, Principal Investigator/Consultant; Dr. Vasileios Tsadlidis, Gateshead Health NHS Foundation Trust, Principal Investigator; Dr. Kilimangalam Narayanan, Gateshead Health NHS Foundation Trust, Co‐Principal Investigator.

## Supporting information


**Table S1.** HRQoL regression outputs for available case and multiple imputation analyses.
**Table S2**. Cost regression outputs for available case and multiple imputation analyses.
