# Supplementary material for: Cost‐effectiveness of swab versus tissue sampling for infected diabetic foot ulcers from the CODIFI2 randomised controlled trial
Source: Diabet Med. 2025 Feb 27;42(5):e15492. doi: 10.1111/dme.15492 (PMC12006556; doi:10.1111/dme.15492)
Supplement: Supplementary file 1 — Table S1. HRQoL regression outputs for available case and multiple imputation analyses. Table S2. Cost regression outputs for available case and multiple imputation analyses. [file DME-42-e15492-s001.docx]

**SUPPLEMENTARY TABLE X (1). HRQoL regression outputs for available case and multiple imputation analyses**

|  | **Survival Analysis** | | | **HRQoL Available Case** | | | **HRQoL Multiple Imputation** | | |
| --- | --- | --- | --- | --- | --- | --- | --- | --- | --- |
|  | Estimate | Std Error | P | Estimate | Std Error | P | Estimate | Std Error | P |
| Intercept 1 | 7.247 | 0.412 | <.0001 | 0.590 | 0.072 | <.0001 | 0.578 | 0.065 | <.0001 |
| Intercept 2 |  |  |  |  |  |  |  |  |  |
| Intercept 3 |  |  |  |  |  |  |  |  |  |
| Mean Adjusted Age | -0.027 | 0.012 | 0.025 | -0.004 | 0.002 | 0.120 | -0.005 | 0.002 | 0.023 |
| Female | 0.283 | 0.390 | 0.468 | -0.072 | 0.066 | 0.281 | -0.061 | 0.055 | 0.265 |
| Not Diabetes Type I | -0.208 | 0.462 | 0.653 | -0.017 | 0.089 | 0.852 | -0.001 | 0.074 | 0.990 |
| Ulcer Area < 1cm^2^ | 0.055 | 0.276 | 0.841 | -0.039 | 0.058 | 0.496 | 0.015 | 0.051 | 0.762 |
| Ulcer Duration < 6 months | 0.367 | 0.335 | 0.274 | -0.028 | 0.066 | 0.674 | -0.023 | 0.054 | 0.669 |
| More Than 1 Ulcer | -0.191 | 0.366 | 0.601 | -0.072 | 0.055 | 0.191 | -0.074 | 0.047 | 0.112 |
| Tissue Sample | 0.453 | 0.268 | 0.092 | 0.038 | 0.060 | 0.521 | 0.026 | 0.061 | 0.673 |
| Tissue Sample * Week 4 |  |  |  | 0.048 | 0.039 | 0.212 | 0.036 | 0.050 | 0.480 |
| Tissue Sample * Week 12 |  |  |  | -0.007 | 0.041 | 0.875 | -0.029 | 0.052 | 0.579 |
| Tissue Sample * Week 26 |  |  |  | 0.037 | 0.043 | 0.382 | -0.013 | 0.054 | 0.816 |
| Tissue Sample * Week 39 |  |  |  | -0.089 | 0.050 | 0.079 | -0.184 | 0.065 | 0.005 |
| Tissue Sample * Week 52 |  |  |  | -0.130 | 0.049 | 0.008 | -0.163 | 0.067 | 0.015 |
| Tissue Sample * Week 104 | |  |  | 0.003 | 0.076 | 0.964 | -0.219 | 0.078 | 0.006 |
| Swab Sample * Week 4 |  |  |  | 0.044 | 0.039 | 0.258 | 0.044 | 0.050 | 0.376 |
| Swab Sample * Week 12 |  |  |  | 0.056 | 0.042 | 0.181 | 0.087 | 0.051 | 0.089 |
| Swab Sample * Week 26 |  |  |  | 0.051 | 0.046 | 0.271 | 0.068 | 0.058 | 0.236 |
| Swab Sample * Week 39 |  |  |  | -0.031 | 0.049 | 0.526 | -0.043 | 0.056 | 0.440 |
| Swab Sample * Week 52 |  |  |  | 0.019 | 0.050 | 0.702 | 0.029 | 0.067 | 0.663 |
| Swab Sample * Week 104 |  |  |  | -0.013 | 0.093 | 0.891 | -0.272 | 0.084 | 0.001 |
| Scale | 0.975 | 0.158 |  |  |  |  |  |  |  |
| Random Effect Variance |  |  |  | 0.072 | 0.010 |  | 0.072 | 0.010 |  |
|  |  |  |  |  |  |  |  |  |  |
| Baseline Difference |  |  |  | 0.038 | 0.060 | 0.521 | 0.026 | 0.061 | 0.673 |
| Week 4 Difference |  |  |  | 0.005 | 0.055 | 0.935 | -0.008 | 0.070 | 0.906 |
| Week 12 Difference |  |  |  | -0.063 | 0.059 | 0.288 | -0.116 | 0.073 | 0.111 |
| Week 26 Difference |  |  |  | -0.014 | 0.063 | 0.825 | -0.081 | 0.079 | 0.306 |
| Week 39 Difference |  |  |  | -0.057 | 0.071 | 0.417 | -0.141 | 0.086 | 0.104 |
| Week 52 Difference |  |  |  | ***-0.149*** | ***0.070*** | ***0.033*** | ***-0.192*** | ***0.090*** | ***0.034*** |
| Week 104 Difference |  |  |  | 0.016 | 0.120 | 0.893 | 0.053 | 0.094 | 0.577 |

HRQoL, health-related quality of life; Std, standard.

**SUPPLEMENTARY TABLE X (2) Cost regression outputs for available case and multiple imputation analyses**

|  | **Probability of antibiotic costs** | | | **Probability of hospitalisation costs** | | | **Other Costs^a^ – Available case** | | | **Other^a^ Costs – Multiple imputation** | | |
| --- | --- | --- | --- | --- | --- | --- | --- | --- | --- | --- | --- | --- |
|  | Estimate | Std Error | P | Estimate | Std Error | P | Estimate | Std Error | P | Estimate | Std Error | P |
| Intercept 1 | -2.847 | 0.126 | <.0001 | -7.380 | 0.406 | <.0001 | 577.63 | 94.38 | <.0001 | 572.60 | 103.43 | <.0001 |
| Intercept 2 |  |  |  | -5.993 | 0.327 | <.0001 |  |  |  |  |  |  |
| Intercept 3 |  |  |  | -4.783 | 0.306 | <.0001 |  |  |  |  |  |  |
| Mean Adjusted Age | -0.006 | 0.003 | 0.075 | -0.005 | 0.007 | 0.507 | 3.44 | 2.88 | 0.234 | 5.00 | 3.51 | 0.155 |
| Female | -0.007 | 0.096 | 0.939 | -0.155 | 0.216 | 0.472 | 101.86 | 81.50 | 0.212 | 112.29 | 90.65 | 0.216 |
| Not Diabetes Type I | 0.122 | 0.116 | 0.295 | 0.011 | 0.253 | 0.966 | 102.34 | 117.81 | 0.386 | 201.11 | 160.86 | 0.213 |
| Ulcer Area < 1cm^2^ | -0.268 | 0.094 | 0.004 | -0.455 | 0.210 | 0.030 | -41.56 | 72.57 | 0.567 | -65.41 | 76.59 | 0.394 |
| Ulcer Duration < 6 months | -0.092 | 0.093 | 0.322 | 0.014 | 0.203 | 0.946 | -38.16 | 83.14 | 0.647 | -57.39 | 103.73 | 0.581 |
| More Than 1 Ulcer | 0.194 | 0.077 | 0.012 | 0.065 | 0.167 | 0.697 | 36.83 | 67.63 | 0.586 | 72.49 | 81.31 | 0.374 |
| Tissue Sample |  |  |  |  |  |  |  |  |  |  |  |  |
| Tissue Sample * Week 4 |  |  |  |  |  |  |  |  |  |  |  |  |
| Tissue Sample * Week 12 | -1.176 | 0.155 | <.0001 | -0.555 | 0.345 | 0.107 | -25.53 | 79.41 | 0.748 | -37.93 | 97.04 | 0.696 |
| Tissue Sample * Week 26 | -1.786 | 0.157 | <.0001 | -1.368 | 0.367 | 0.000 | -81.94 | 80.95 | 0.312 | -59.50 | 96.42 | 0.537 |
| Tissue Sample * Week 39 | -2.107 | 0.178 | <.0001 | -1.733 | 0.418 | <.0001 | -177.60 | 89.97 | 0.049 | -178.38 | 102.46 | 0.082 |
| Tissue Sample * Week 52 | -1.946 | 0.171 | <.0001 | -1.495 | 0.393 | 0.000 | -83.69 | 90.06 | 0.354 | -98.80 | 106.77 | 0.355 |
| Tissue Sample * Week 104 | -2.208 | 0.142 | <.0001 | -1.769 | 0.337 | <.0001 | -270.45 | 131.26 | 0.040 | -234.29 | 191.00 | 0.222 |
| Swab Sample * Week 4 | -0.230 | 0.149 | 0.122 | -0.381 | 0.397 | 0.337 | -42.77 | 88.98 | 0.631 | -32.61 | 100.56 | 0.746 |
| Swab Sample * Week 12 | -1.473 | 0.173 | <.0001 | -0.989 | 0.387 | 0.011 | -160.81 | 94.33 | 0.089 | -161.71 | 106.82 | 0.130 |
| Swab Sample * Week 26 | -1.880 | 0.167 | <.0001 | -1.041 | 0.344 | 0.003 | -195.07 | 100.17 | 0.052 | -190.16 | 107.21 | 0.076 |
| Swab Sample * Week 39 | -1.954 | 0.176 | <.0001 | -1.323 | 0.378 | 0.001 | -181.32 | 107.06 | 0.091 | -121.12 | 116.12 | 0.297 |
| Swab Sample * Week 52 | -2.548 | 0.224 | <.0001 | -1.877 | 0.456 | <.0001 | -291.69 | 105.06 | 0.006 | -278.73 | 118.95 | 0.020 |
| Swab Sample * Week 104 | -2.114 | 0.146 | <.0001 | -1.462 | 0.327 | <.0001 | -55.71 | 155.23 | 0.720 | -32.57 | 216.23 | 0.881 |
| Scale |  |  |  |  |  |  |  |  |  |  |  |  |
| Random Effect Variance |  |  |  |  |  |  | 69613 | 17789 |  | 69613 | 17789 |  |
|  |  |  |  |  |  |  |  |  |  |  |  |  |
| Baseline Difference |  |  |  |  |  |  |  |  |  |  |  |  |
| Week 4 Difference | 0.230 | 0.149 | 0.122 | 0.381 | 0.397 | 0.337 | 42.77 | 88.98 | 0.631 | 32.61 | 100.56 | 0.746 |
| Week 12 Difference | 0.298 | 0.187 | 0.112 | 0.434 | 0.377 | 0.250 | 135.28 | 97.49 | 0.166 | 123.78 | 103.13 | 0.230 |
| Week 26 Difference | 0.094 | 0.183 | 0.606 | -0.327 | 0.356 | 0.359 | 113.13 | 105.44 | 0.284 | 130.66 | 110.68 | 0.238 |
| Week 39 Difference | -0.154 | 0.210 | 0.464 | -0.410 | 0.437 | 0.349 | 3.72 | 118.15 | 0.975 | -57.26 | 120.85 | 0.636 |
| Week 52 Difference | ***0.602*** | ***0.246*** | ***0.014*** | 0.382 | 0.486 | 0.432 | 208.00 | 116.42 | 0.075 | 179.93 | 133.68 | 0.179 |
| Week 104 Difference | -0.094 | 0.151 | 0.531 | -0.307 | 0.307 | 0.317 | -214.74 | 188.83 | 0.256 | -201.72 | 252.64 | 0.426 |

Other = Sampling, HCP contacts and non-admitted care; Std, standard
